# Preface—Plant Proteomic Research

**DOI:** 10.3390/ijms18010088

**Published:** 2017-01-04

**Authors:** Setsuko Komatsu, Zahed Hossain

**Affiliations:** 1 National Institute of Crop Science, National Agriculture and Food Research Organization, Kannondai 2-1-18, Tsukuba 305-8518, Japan; 2 Department of Botany, University of Kalyani, West Bengal 741235, India

Plants, being sessile in nature, are constantly exposed to environmental challenges resulting in substantial yield loss. To cope with harsh environments, plants have developed a wide range of adaptation strategies involving morpho-anatomical, physiological, and biochemical traits. In recent years, there has been phenomenal progress in the understanding of plant responses to environmental cues at the protein level. This progress has been fueled by the advancement in mass spectrometry techniques, complemented with genome-sequence data and modern bioinformatics analysis with improved sample preparation and fractionation strategies. As proteins ultimately regulate cellular functions, it is perhaps of greater importance to understand the changes that occur at the protein-abundance level, rather than the modulation of mRNA expression. This special issue on “Plant Proteomic Research” brings together a selection of insightful papers that address some of these issues related to applications of proteomic techniques in elucidating master regulator proteins and the pathways associated with plant development and stress responses. This issue includes four reviews and 13 original articles primarily on environmental proteomic studies.

The first review by Hossain et al. [1] summarizes the recent contributions of plant proteomic research to comprehend the complex mechanism of plant response to nanoparticles stress. Pechanova and Pechan [2] present an overview of maize-pathogen interactions at the proteome level, emphasizing the application of various mass spectrometry-based high-throughput proteomic techniques in identifying possible candidate proteins involved in maize pathogen resistance. Wang et al. [3] summarize the recent proteomic studies related to drought sensing and signaling mechanisms for better understanding the molecular basis of plant drought tolerance. Nouri et al. [4] present a comprehensive picture about the fine tuning of photosynthetic pathways at the protein level linked to plant adaptation to abiotic stresses.

Among the 13 original articles, six articles highlight iTRAQ-based proteomic approaches. Ye et al. [5] present a deep and extensive research work on drought-induced leaf proteome modulation in Switchgrass (*Panicum virgatum*) using the iTRAQ labeling method followed by nano-scale liquid chromatography mass spectrometry analysis. Li et al. [6] provide an overview of aluminum stress-mediated alterations of leaf proteome in two contrasting citrus species differing in aluminum tolerance. An et al. [7] present comparative proteomic analysis of ramie plants under PEG-mediated drought stress. Hua et al. [8] emphasize the application of proteomic analysis in unraveling the molecular mechanism of betalain biosynthesis in *Hylocereus polyrhizus* fruits at the posttranscriptional level. Li et al. [9] present comparative proteomic analyses of buds and young expanding leaves of the tea plant (*Camellia sinensis* L.), highlighting the molecular mechanism involved in secondary metabolite production. Zhao et al. [10] perform quantitative proteomics analysis of herbaceous peony (*Paeonia lactiflora* Pall.) in response to Paclobutrazol, a triazole compound inhibiting growth of lateral branching. By using gel-free proteomics, Alqurashi et al. [11] analyze the changes in the *Arabidopsis thaliana* proteome composition implicating the role of cAMP in biotic and abiotic stress responses by inducing complex changes in cellular energy homeostasis. Rasool et al. [12] present the comprehensive expression pattern of peptides in the date palm stem infested with Red Palm Weevil (*Rhynchophorus ferrugineus* Oliv.) using two-dimensional gel electrophoresis (2-DE) and MALDI-TOF mass spectrometry. Liu et al. [13] provide new insights into the molecular regulation of leaf color variation and carbon fixation in a xantha mutant of *Ginkgo biloba* L. by exploiting 2-DE coupled with MALDI-TOF/TOF mass spectrometry. Yu et al. [14] present comparative proteomic analysis of chimera *Hosta* “Gold Standard” leaves from various regions at different development stages and under excess nitrogen fertilization using 2-DE coupled MALDI-TOF/TOF MS. Findings provide new insights towards understanding the mechanisms of leaf color regulation in variegated leaves. Zhang et al. [15] unravel the protein regulation mechanism of pollen infertility and allergenicity in triploid and diploid poplar (*Populus deltoids*) plants using the 2-DE technique followed by MALDI-TOF-TOF mass spectrometry analysis. Wang et al. [16] demonstrate the stable expression of basic fibroblast growth factor in chloroplasts of tobacco providing an additional option for the production of chloroplast-produced therapeutic proteins. Integration of the foreign expression cassette into the plastid genome of transformants is confirmed by PCR and Southern hybridization and expression is quantified by ELISA. Liu et al. [17] provide an overview of the seed-specific expression of microtubule-associated protein SBgLR in transgenic maize (*Zea mays*), resulting in increased seed protein and lysine contents. The zein, non-zein, and total protein extracts of the seeds of transgenic plants are analyzed by sodium dodecyl sulfate-polyacrylamide gel electrophoresis (SDS-PAGE).

This special issue on “Plant Proteomic Research” is an attempt to provide researchers with a glimpse of advanced mass spectrometry techniques with a special emphasis on candidate proteins and pathways associated with plant development and stress responses. We believe that this special issue reflects the current perspective and state of the art of plant proteomics, which would not only enrich us in understanding the plant’s response to environmental clues but would further help us in designing better crops with the desired phenotypes. The articles in this issue will be of general interest to proteomic researchers, plant biologists, and environmental scientists.

We would like to express our gratitude to all authors for their high quality contributions and numerous peer reviewers for their critical evaluation and valuable suggestions. Moreover, we render our heartiest thanks to the Managing Editor Yong Ren and Section Managing Editor Yue Chen for giving us the opportunity to serve “Plant Proteomic Research” as Guest Editors and Editorial Office, a special mention goes to Sophie Suo for her untiring efforts in coordinating with authors and keeping us updated about the manuscript submission and review process, which helped us in completing the surmount task on time. Finally, we extend our sincere thanks to those professionals whose expertise in proofreading and formatting greatly improved the quality of this special issue.
